# The relationship between sleep quality and road traffic crashes of urban drivers in Hamadan, Iran

**DOI:** 10.5249/jivr.v12i1.1262

**Published:** 2020-01

**Authors:** Roya Amini, Forouzan Rezapur-Shahkolai, Masoud Khodaveisi, Shirin Gorjian, Ali Reza Soltanian

**Affiliations:** ^*a*^ Chronic Diseases (Home care) Research Center, Department of Community Health Nursing, School of Nursing and Midwifery, Hamadan University of Medical Sciences, Hamadan, Iran.; ^*b*^ Department of Public Health, School of Public Health & Research Center for Health Sciences, Hamadan University of Medical Sciences, Hamadan, Iran.; ^*c*^ Chronic Diseases (Home care) Research Center, Department of Community Health Nursing, School of Nursing and Midwifery, Hamadan University of Medical Sciences, Hamadan, Iran.; ^*d*^ Department of Community Health Nursing, School of Nursing and Midwifery, Hamadan University of Medical Sci-ences, Hamadan, Iran.; ^*e*^ Modeling of Non-communicable Diseases Research Center, Department of Biostatistics, School of Health, Hamadan University of Medical Sciences, Hamadan, Iran.

**Keywords:** Sleep hygiene, Road traffic crashes, Automobile driving, Cities

## Abstract

**Background:**

Sleep quality is one of the main human factors related to urban road traffic crashes. This study aimed at determining the relationship between sleep quality and road traffic crashes in urban drivers.

**Methods:**

This correlational study was conducted in Hamadan, a city located in the western part of Iran. The study samples consisted of 309 Hamadan drivers (i.e., 103 with road traffic crashes (RTCs) and 206 without RTCs), who were referred to police centers to change or renew their driving licenses. The data collection tool was a two-part questionnaire including demographic information and the Pittsburgh Sleep Quality Index (PSQI). The questionnaire was filled out in a self-administered manner. Statistical analysis was done using the SPSS-16 software and applying logistic regression, Fisher’s exact test, and Chi-square test.

**Results:**

The comparison of sleep quality scores between two groups, using the adjusted logistic regression test, showed a statistically significant difference between them (P = 0.019). This means that the sleep quality of drivers without RTCs was 1.8 times better than drivers with RTCS (OR=1.8; 95% CI, 1.1 - 3.07).

**Conclusions:**

There was a significant association between poor sleep quality and the occurrence of RTCs in urban drivers. As a result, it is recommended paying more attention to the sleep quality of urban drivers to prevent and control RTCs.

## Introduction

Car crashes are an unfortunate event that occurs unpredictably and involuntarily, typically resulting in damage.^1^ According to “the global status report” of the World Health Organization (WHO) on road safety in 2015, there were 1.25 million road traffic deaths globally,^2^ 90% of which occurred in low-income and middle-income countries.^3^ Iran is among the countries with high road traffic crashes (RTCs),^4^ which is a leading cause of death and disability among the Iranian population.^5^ It is estimated that more than 20,000 to 80,000 individuals in Iran have been killed or injured due to RTCs each year.^4^

Human factors play a key role in the occurrence of RTCs.^6^ Sleep deprivation is one of the main human factors that can affect driving and lead to RTCs.^7^ The most common result of RTCs in urban areas of Iran is related to the human factors. In addition, sleep deprivation has been identified as the most crucial risk factor in this regard.^8^ Some studies have shown that as a consequence of drowsiness, the probability of RTCs may increase by 1.6-3.8%.^9^

Even in some studies conducted in the United States, France, and New Zealand, the possibility of RTCs exceeds this figure with the statistics ranging from 3.9% to 33%.^10-12^ Accordingly, it can be stated that sleep deprivation can lead to motor vehicle crash death. According to a study, the reason for 10%-20% of RTCs fatalities is sleep deprivation during driving.^11^

Based on the Pittsburgh Sleep Quality Index (PSQI), sleep quality is comprised of seven components: subjective sleep quality, sleep latency, sleep duration, habitual sleep efficiency, sleep disturbances, use of sleeping medications, and daytime dysfunction over the last month.^13^ Sleep problems may be due to a variety of reasons such as sleep-related illnesses like sleep apnea; lifestyle factors; and occupational characteristics such as rotating shift schedules.^11^ According to some studies, the risk of RTCs increases in people who sleep less than 6 hours per day.^14^ Moreover, concentration in driving significantly declines in people working at night shift such as nurses and other hospital workers.^15^ Furthermore, the risk of RTCs increases in people suffering from poor-quality sleep and taking sleeping medication. ^16^

To prevent RTCs, providing adequate information on the causes of RTCs is essential. The rate of RTCs is growing and sleep quality has been considered the most significant human factor in countries such as Iran.^17^ In this regard, a limited number of studies have been performed to determine the association between sleep deprivation and RTCS in urban areas. Therefore, this study aimed at determining the relationship between sleep quality and RTCs of drivers in Hamadan.

## Methods 

This correlational study was conducted in Hamadan, a city located in the western part of Iran, in 2013. The participants were (cars, taxies, and van) drivers referred to police centers (known in Iran as Police+10) to renew or replace their driving license. It is noteworthy that in Iran, many jobs like issuing and renewing driving licenses are done by Police+10 centers. The sample size was calculated as 309 drivers using a 40% prevalence of sleep quality impairment, ^18^ 80% power with a 2-sided significance level of α = 0.05, and the effect size of 0.70.

The sampling was done in two steps. First, 4 centers out of 6 Police+10 centers (more than 60% of Police+10 centers) were selected randomly and then drivers from each center were chosen by the convenience sampling method. After that, the samples were divided into two groups consisting of drivers who have experienced RTCs (n=103) and those who have not experienced RTCs (n= 206), in the city of Hamadan during the last year. The inclusion criteria were having the age range between 18 to 65 years; reading and writing skills in responding to the self-administered questionnaires; residing in the city of Hamadan; and having a driver’s permit for both groups.

Data Collection: after obtaining permission from the Deputy of Research and Technology of Hamedan University of Medical Sciences, the researcher was referred to the Police+10 station in Hamadan. The drivers were asked about the occurrence of RTCs during the last year. Consequently, they were divided into drivers involved in RTCs and those with no RTCs. The participants were given a brief explanation of the research and how to respond to the questionnaire. In addition, the participants declared their voluntary and informed consent to participate in the research. Concurrent sampling was carried out in both groups from October to December 2013.

The study tool was a two-part questionnaire including demographic information (i.e., gender, marital status, age, education level, occupation, monthly income, and the number of family members) and the PSQI. The PSQI was designed in 1989 by Daniel Jay Bice and colleagues and was revised in 2000. This self-reported questionnaire assesses sleep quality and disturbances over a one-month time interval. This questionnaire consists of 7 components including subjective sleep quality, sleep latency, sleep duration, habitual sleep efficiency, sleep disturbances, use of sleeping medication, and daytime dysfunction. Each component takes a score ranging from 0 to 3. The scores of 0, 1, 2, and 3 in each component represents the normal state, the presence of a mild, moderate, and severe problem, respectively. The Global PSQI Score measures by adding the seven component scores together and giving a whole mark ranging from 0 to 21. The total score of five or more means an inappropriate quality of a person’s sleep and a score of less than five is a sign of the appropriate sleep quality. The internal consistency (Cronbach’s α = 0.83) and reliability (Cronbach’s α = 0.81) of the PSQI questionnaire were confirmed^19,20^ in a study in Iran.^21^

Data analysis was done using SPSS-16 software. Analytical tests used in the study to assess the sleep quality in both groups consisted of a logistic regression test, Chi-square test, and Fischer’s exact test. The level of significance was considered to be a p-value of less than 0.05 (P<0.05).

This study was approved by the Ethics Committee of Hamadan University of Medical Sciences (No.: 920321823).

## Results

According to the obtained results, the mean ages of the drivers with the experience of RTCs and without this experience were 29.8 ± 8.88 and 32.47 ± 8.60 years, respectively. The highest number of participants in the RTCs group was in the range of 31 to 35 years old (37.4%), and 30.1% were in the range of 26 to 30 in the without RTCs group. The results showed that there was a statistically significant difference between the two groups regarding age distribution (P = 0.018). Moreover, 80.6% of the RTCs drivers and 80.1% of drivers without RTCs were male. Comparing the results, there is no significant difference in gender distributions in two groups (P = 0.92). Most of the drivers with the experience of RTCs (28.2%) were graduated from high school, but most drivers who did not RTCs experience (50%) had undergraduate studies (P = 0.001) (Table 1).

**Table 1:** Demographic information of drivers with and without RTCs.

Variable	Classification	Without RTCs N (%)	With RTCs N (%)	Statistical Test
Age (year)	19-25	42 (20.4%)	13 (12.6%)	X^2^=17.8
	26-35	118 (57.3%)	53 (51.5%)	P= 0. 018
	36-45	26 (12.5%)	23 (22.3%)	
	>45	20 (9.8%)	14 (13.6%)	
Gender	Male	165 (80.1%)	83 (80.6%)	X^2^=0.1
	Female	41 (19.9%)	20 (19.4%)	P= 0.92
Education	Elementary	5 (2.4%)	2 (1.9%)	X^2^=12.4
	Intermediate	9 (4.4%)	13 (12.6%)	p=0.001
	Diploma	13 (15%)	30 (29.2%)	
	Post Diploma	58 (23.8%)	21 (20.4%)	
	Bachelor and higher	121 (54.4%)	37 (35.9%)	
Occupation	Worker	2 (1%)	7 (6.8%)	X^2^= 41.43
	Employee	63 (30.5%)	31 (30.1%)	P<0.001
	Self-employment	22 (10.7%)	34 (33%)	
	Unemployed	89 (43.3%)	15 (14.5%)	
	Housekeeper	30 ( 14.5%)	16 15.6%)	

RTCs: Road Traffic Crashes X2 = Chi-square Test

The results showed that over the past one year, 144 drivers without the RTCs had a good sleep quality in the recent month (69.8%); while at the same time, among those who had RTCS, only 47 drivers (45.6%) had a good sleep quality. According to age and gender-adjusted logistic regression analysis, there were no similarities between the sleep quality for neither of groups (P= 0.019; OR=1.8; 95%CI = 1.1-3.07). This means that the sleep quality of drivers without the RTCs was 1.8 times better than individuals with the occurrence of RTCs (Table 2).

**Table 2:** Adjusted logistic regression analysis for comparing global sleep quality in two groups of drivers.

Level of Sleep Quality	Without RTCs N (%)	With RTCs N (%)	CI =%95 Upper Lower
Weak	62 (30.2%)	56 (54.4%)	B=0.610
(>5)			SE= 0.260
Good	144 (69.8%)	47 (45.6%)	P value= 0.019
(<5)			OR=1.841
whole	206 (100%)	103 (100%)	CI 95%= 1.1, 3.07

Adjusted: age and gender

Regarding the seven components of sleep quality, there was no significant statistical difference between drivers with and without the RTCs in terms of subjective sleep quality (P=0.120), sleep latency (P=.0289), sleep duration (P=0.403), habitual sleep efficiency (P = 0.507), sleep disturbances (P= 0.13), use of sleeping medication (P=0.667), and daytime dysfunction (P= 0.073) (Table 3). 

**Table 3:** Sleep quality components of drivers with and without RTCs.

Components of sleep quality	Classification	Without RTCs* N (%)	With RTCs N (%)	Statistical test
Subjective sleep quality	Very good	50 (24.3%)	17(16.5%)	P=0.209
	Fairly Good	128(62.1)	65(63.1)	**X^2^=4.301
	Fairly bad	27(13.1)	20(19.4)	
	Very bad	1(0.5)	1(1)	
Sleep latency (minutes)	≤15	91(44.2)	35(34)	P=0.289
	15-30	85(41.3)	51(49.5)	**X^2^=4.902
	31-60	23(11.2)	13(12.6)	
	>60	7(3.4)	4(3.9)	
Sleep duration (hours)	>7	134(65)	60(58.3)	P=0.403
	6-7	50(24.3)	28(27.2)	**X^2^=2.893
	5-6	18(8.7)	10(9.7)	
	>5	4(1.9)	5(4.9)	
Habitual sleep efficiency(%)	%85>	204(99)	101(98.1)	P=0.507
	%75-84	2(1)	1(1)	***X^2^=2.056
	%65-74	0(0)	0(1)	
	%65<	0(0)	1(1)	
Sleep disturbances	Not during the past month	17(8.3)	7(6.8)	P=0.113
	Less than once a week	174(85.5)	82(79.6)	**X^2^=6.406
	Once or twice a week	14(6.8)	14(13.6)	
	Three or more times a week	1(0.5)	0(0)	
Use of sleeping medication	Not during the past month	177(85.9)	84(81.6)	P=0.667
	Less than once a week	21(10.2)	14(13.6)	***X^2^=1.688
	Once or twice a week	6(2.9)	3(2.9)	
	Three or more times a week	2(1)	2(1.9)	
Daytime dysfunction	Never	99(48.1)	34(33)	P=0.073
	Once or twice	91(44.2)	60(58.3)	**X^2^=6.690
	Once or twice each week	12(5.8)	7(6.8)	
	Three or more times each week	4(1.9)	2(1.9)	

*RTCs: Road Traffic Crashes **X2 = Chi-square Test *** X2 = Fisher’s exact test

## Discussion

This study showed that the global sleep quality for the majority of drivers (69.8%) without the experience of RTCs fell within the category of “good” sleepers (sleep quality score was less than 5). Furthermore, in the drivers who did not have a record of RTCs over the previous year, the sleep quality was 1.8 times better than drivers with the record of RTCs. Therefore, there was a statistically significant relation between poor sleep quality and the occurrence of RTCs in drivers.

There is a similarity between the finding of this study and the one conducted by Hojjati et al. (2010) regarding bus drivers in urban areas of Iran. They examined the sleep quality of all drivers and found that sleep quality for drivers was strongly associated with the occurrence of RTCs.^22^

In this study, the subjective sleep quality was slightly better, but not significant, in drivers without RTCs record compared to the drivers with the record of RTCs. This result is somewhat consistent with the study of Malek et al. in Iran; they found that about one-third of all the crashes were related to the drivers’ sleep quality.^23^ It is noteworthy that in the study of Malek et al., RTCs were considered in heavy truck drivers on the roads, in whom longer driving, fatigue, and sleep deprivation increase the likelihood of driving accidents.

Another component of sleep quality affected by RTCs is sleep latency, which was 15-30 minutes and less than 15 minutes for most of the drivers with and without the experience of RTCs, respectively. It can be concluded that drivers with the RTCs record had more difficulty falling asleep, but there was not a significant difference between the two groups in terms of sleep latency. In this regard, Shapiro et al. have shown that objective sleep deprivation like sleep latency affects driving performance,^24^ leading to an increase in the chance of RTCs

One of the components of sleep quality is sleep duration. In the present study, the proportion of drivers with components more than 7 hours of sleep was different from drivers with the RTCs record and those without RTCs record had a slightly longer sleep duration, although not significantly different. This finding is similar to some Japanese^25^ and Iranian studies^26^ claiming fewer sleep hours leads to road traffic crashes. According to Badawys’ study in Egypt, the mean sleep duration was less in those with the record of RTCs compared to those without the record of RTCs.^27^

In this study, the sleep disturbances were less frequent (however, not significant) in drivers without RTCs record compared to those with the record of RTCs. The results of this study are consistent with Hermann’s study. Herman et al. (2013), in their case-control study on motorcyclists in Fiji (Oakland-New Zealand), found that the number of RTCS in drivers who had symptoms of drowsiness was 6 times more than the conscious drivers.^28^ Komada et al. (2013), in their review study on Japanese drivers, reported that sleep deprivation is an important factor in traffic accidents.^25^ According to Philip et al., sleepiness at the wheel and sleep disorders are the main cause of traffic accidents in highway drivers of French.^29^ Phillips and et al. (2013) in a study on drivers in Oslo-Norway^30^ and Pizza and et al. (2010) in Italy have concluded that sleep disorders, sleep deprivation, and severe insomnia during the day resulted in driving accidents.^31^

We also assessed using sleeping medication. In this study, the use of sleeping medication during the past month was more frequent (however, not significant) in the drivers with RTCs record compared to the drivers without the RTCs. The results of this study are consistent with those of Moradi et al., who reported that the risk of driving accidents in drivers taking drugs is slightly higher than other people in Iran.^26^

Another key finding of this study was the relation between daytime dysfunction and RTCs. In the present study, the majority of drivers who did not experience RTCs claimed that they have never experienced any daytime dysfunction during the last month; whereas it has been experienced once or twice a month by the most drivers with RTCs records. The results of this study are consistent with studies related to drivers in the United States, in which the sleep quality of the drivers was associated with job performance and concentration.^32^ Lim et al. also reported an association between daytime driver fatigue a very poor or poor self-rating sleep quality. ^33^

An important limitation of this study was that the sample population was selected among drivers who referred to the Police+10 offices and the other drivers who did not need to change their driving permits were not included. Another limitation of this study was the self-reporting data gathering manner. However, to prevent recall bias, the Pittsburgh questionnaire was used because it assesses the sleep quality retrospectively over a month period.

## Conclusion

According to the results of this study, there was a significant association between sleep quality and records of RTCs in urban drivers. Furthermore, drivers who did not experience RTCs over the previous year had a 1.8-time better sleep quality than the drivers with the record of RTCs. The other outcome of this research was that the seven components of sleep quality (i.e., subjective sleep quality, sleep latency, sleep duration, daytime dysfunction, sleep disturbances, use of sleeping medication, habitual sleep efficiency) were slightly better, although not significantly different in drivers without RTCs record compared to the drivers with the record of RTCs. Therefore, to prevent urban RTCs, it is recommended paying much attention to the sleep quality of urban drivers.

**Acknowledgments**

This study, as a part of the thesis, was approved and supported by the Deputy of Research and Technology of the Hamadan University of Medical Sciences. The authors would like to thank all those who participated in this study.
